# Performance Analysis of Self-Collected Nasal and Oral Swabs for Detection of SARS-CoV-2

**DOI:** 10.3390/diagnostics12102279

**Published:** 2022-09-21

**Authors:** Ho-Jae Lim, Young-Hyun Baek, Min-Young Park, Jae-Hyun Yang, Min-Jin Kim, Nackmoon Sung, Yong-Hak Sohn, Sun-Hwa Lee, Jung-Eun Park, Yong-Jin Yang

**Affiliations:** 1 Department of Molecular Diagnostics, Seegene Medical Foundation, Seoul 04805, Korea; 2 Department of Integrative Biological Sciences & BK21 FOUR Educational Research Group for Age-Associated Disorder Control Technology, Chosun University, Gwangju 61452, Korea; 3 Paul F. Glenn Center for Biology of Aging Research, Department of Genetics, Blavatnik Institute, Harvard Medical School, Boston, MA 02115, USA; 4 Clinical Research Institute, Seegene Medical Foundation, Seoul 04805, Korea; 5 Department of Laboratory Medicine, Seegene Medical Foundation, Seoul 04805, Korea

**Keywords:** SARS-CoV-2, mRT-qPCR, HCW-collection, self-collection, large-scale sampling, viral load

## Abstract

Severe acute respiratory syndrome coronavirus 2 (SARS-CoV-2) is the third highly pathogenic human coronavirus and is rapidly transmitted by infected individuals regardless of their symptoms. During the COVID-19 pandemic, owing to the dearth of skilled healthcare workers (HCWs) to collect samples for early diagnosis, self-collection emerged as a viable alternative. To evaluate the reliability of self-collection, we compared the virus detection rate using 3990 self-collected swabs and HCW-collected swabs, procured from the same individuals and collected immediately after the self-collection. The results of multiplex reverse-transcription quantitative polymerase chain reaction revealed that the viral load in the HCW-collected swabs was marginally (18.4–28.8 times) higher than that in self-collected swabs. Self-collection showed no significant difference in sensitivity and specificity from HCW-collection (κ = 0.87, McNemar’s test; *p* = 0.19), indicating a comparable performance. These findings suggest that self-collected swabs are acceptable substitutes for HCW-collected swabs, and that their use improved the specimen screening efficiency and reduced the risk of SARS-CoV-2 infection among HCWs during and after the COVID-19 pandemic.

## 1. Introduction

Severe acute respiratory syndrome coronavirus 2 (SARS-CoV-2) is the third highly pathogenic human coronavirus to emerge in recent years [1]. This novel coronavirus was initially identified in December 2019; the World Health Organization declared the coronavirus disease 2019 (COVID-19) a global pandemic on March 11, 2020 [2]. Currently, vaccination is the most effective strategy for combating the pandemic; however, viral evolution (driven by genomic mutations) threatens the efficacy of vaccines [3]. Despite multiple vaccinations, breakthrough SARS-CoV-2 infections have become common owing to the emergence of variants, including Delta (B.1.617.2) and Omicron (B.1.1.529) [4,5]. Despite increased screening and testing, more than 551 million confirmed cases and 6.3 million deaths had been documented worldwide by July 11, 2022 [6].

SARS-CoV-2 is transmitted through both symptomatic and asymptomatic carriers [7]. Therefore, early diagnosis of positive cases significantly reduces the virus spread [8]. The virus has been detected in numerous clinical specimens, including nasopharyngeal swabs (NPS), oropharyngeal swabs (OPS), nasal swabs (NS), and oral swabs (OS); as well as in sputum, urine, stools, and blood [9,10]. Current surveillance relies on established sampling techniques, such as the collection of NPS by trained healthcare workers (HCWs) [11,12,13]. However, a shortage of HCWs has strained the NPS sampling system, especially when the incidence of infections increases rapidly [14]. The Food and Drug Administration authorized rapid antigen tests for over-the-counter usage in December 2020 [15]. However, the sensitivity of these antigen tests is much lower than that of the polymerase chain reaction (PCR) test; therefore, cases need to be validated by PCR [16]. Consequently, it is necessary to expand testing capacity, including self-collection of respiratory specimens (NS, OS, and saliva), for PCR testing [10].

HCWs collect NPS and OPS specimens by swabbing the posterior nasopharynx and oropharynx, respectively [17]. Self-collection of NS and OS specimens can be performed simply by swabbing the anterior nares and mouth [10]. Numerous investigations have evaluated the accuracy of SARS-CoV-2 test results from self-collected vs. HCW-collected specimens. Some studies reported no significant difference between the two collection methods in terms of diagnostic sensitivity (86.3–89.2%) [18,19,20]; however, a low reliability of test results from self-collected samples was reported in some other studies [21].

Because sampling by HCWs is limited by time and place [22], previous findings have suggested that the time delay between disease onset and sampling may be variable. The diagnostic performance of self-collected swabs has been evaluated using small sample sizes; however, large-scale (≥ 3000) sampling has not yet been conducted. Therefore, research on the diagnostic efficacy of self-collected swabs is crucial for the prevention of disease transmission and early diagnosis. In this study, we assessed the detection rate and viral load using large-scale sampling and evaluated the diagnostic performance of the self-collection system to highlight its potential as an alternative to collection by HCWs. The results demonstrate that self-collection could replace HCW-collection for the diagnosis of SARS-CoV-2.

## 2. Materials and Methods

### 2.1. Study Design and Inclusion and Exclusion Criteria

This study was designed as a collaboration between Ewha Womans University and Seegene Medical Foundation for the Ewha Safe Campus (ESC) project. This project was developed by the university safety campus management as a model for the SARS-CoV-2 infection. The study was approved by the Institutional Review Board of the Seegene Medical Foundation (SMF-IRB-2022-022). Informed consent from the participants was waived because the data collected for this study were anonymized. Anonymized data were obtained from the ESC project between February and April 2022. A total of 16,478 participants were enrolled in the study and divided into two groups: (i) both self-collection and HCW-collection (3990 participants), and (ii) either self-collection or HCW-collection (12,488 participants). Participants from the latter group were excluded from this study. The participants were provided visual instructions according to the self-collection manual (Korean version). Self-collection was first conducted under the supervision of HCWs, and HCW-collection was conducted immediately thereafter. Self-collection was performed by first swabbing the anterior nares and then the mouth, whereas HCW-collection was performed by swabbing the oropharynx first and then the posterior nasopharynx. Each sample was analyzed immediately after collection. The outcomes of the test using self-collected samples were compared with those of the HCW-collected samples.

### 2.2. Collection and Experimental Protocol

HCW-collection was used as the gold standard: combined NPS and OPS swabs were collected in ALLTM medium (SG Medical, Seoul, Republic of Korea). The NS and OS swabs were self-collected in SELTM medium (SG Medical). Based on the collection site, ALLTM and SELTM universal transport media were classified into two sets of swabs. Nucleic acids were simultaneously extracted from HCW- and self-collected specimens using the automated MagNA Pure 96 system (Roche, Inc., Basel, Switzerland), as described previously [23]. Following the Pathogen Universal 200 protocol, 200 µL of the samples was processed using magnetic beads for nucleic acid extraction. The purified nucleic acids were eluted in 100 µL of elution buffer.

### 2.3. Multiplex Reverse Transcription Quantitative Real-Time Polymerase Chain Reaction (mRT-qPCR) Analysis

mRT-qPCR was performed using the Allplex^TM^ SARS-CoV-2 Assay kit (Seegene Inc., Seoul, Republic of Korea) to detect the SARS-CoV-2 genes encoding the envelope (*E*), RNA-dependent RNA polymerase (*RdRP*), spike protein (*S*), and nucleocapsid (*N*), following a recent study [24]. If more than one of the target genes (*E*, *RdRP* and *S*, and *N*) were not detected in HCW-collected samples, the result was considered negative [25]. Positive samples were categorized as weakly positive (30–40 Ct), moderately positive (20–30 Ct), or strongly positive (10–20 Ct).

### 2.4. Viral RNA Load Standard

The nucleic acid of the SARS-CoV-2 strain NCCP-43330 was procured as an RNA powder from the National Culture Collection for Pathogens (NCCP; Cheongju, South Korea). The viral RNA was serially diluted in Tris-EDTA buffer to 10^3^ copies/mL for use as a quantification standard. The viral RNA load was measured in triplicates from 10^9^ to 10^3^ copies/mL (Supplementary Figure S1). Raw Ct values of HCW- and self-collected samples were converted to viral RNA loads based on the SARS-CoV-2 standard curve, using the following formula: Viral load = 10^(Ct value − 47.01)/−3.40^ for *N*, 10^(Ct value − 47.79)/−3.39^ for *RdRP* and *S*, and 10^(Ct value − 46.35)/−3.45^ for *E*.

### 2.5. Statistical Analysis

All statistical analyses were performed using R Studio (version 4.1.2; R_Studio Inc., Boston, MA, USA). Categorical variables (positive or negative) are reported in percentages, absolute numbers, and 95% confidence intervals (CI). We defined the detection rate as the ratio of positivity for either self- or HCW-collection among self- and HCW-collected samples with at least one positive. The performance of a diagnostic test for SARS-CoV-2 was evaluated by estimating the sensitivity and specificity for self-collected samples against HCW-collected samples using the epiR package. Using Cohen’s kappa (κ) and McNemar’s significance test from the caret package, perfect concordance (κ = 1), almost perfect agreement (κ > 0.9), or strong agreement (κ > 0.8) was determined between HCW-collected and self-collected samples [26]. The McNemar’s test was used to evaluate the reliability of paired differences (false positives and false negatives) between the two collection methods [27]. A paired *t*-test was used to determine the difference in Ct values between the HCW- and self-collection groups. Results were considered statistically significant at *p* < 0.05. Continuous variables of the mRT-PCR Ct values are represented by mean or median (interquartile range (IQR)). The scatter, histogram, and box plots were generated using the ggplot2 packages.

## 3. Results

### 3.1. Characteristics of the Study Participants

The demography and characteristics of the 3990 eligible participants are presented in Table 1. Samples from the 3990 participants were collected immediately and classified into self- or HCW-collection groups. The percentages of participants showing SARS-CoV-2-positive and -negative results in the self-collection group were 23.9% (*n* = 954/3990) and 76.1% (*n* = 3036/3990) and those in the HCW-collection group were 23.4% (*n* = 935/3990) and 76.6% (*n* = 3055/3990), respectively. The Ct values for the *N*, *RdRP* and *S*, and *E* were similar in each group.

### 3.2. Comparison of the Clinical Diagnosis Performance between the HCW- and Self-Collected Samples

**Figure 1:**
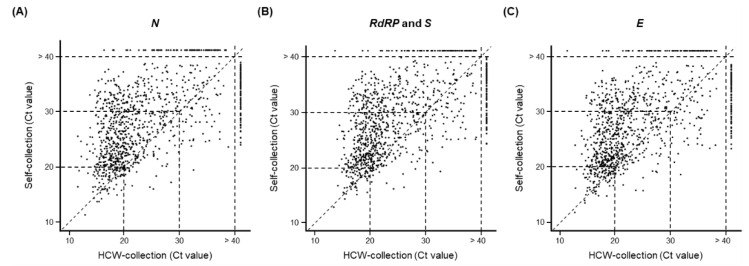
Comparison of Ct values for three SARS-CoV-2 genes detected in 1039 self- and HCW-collected samples that were positive for SARS-CoV-2. (**A**) *N*, (**B**) *RdRP* and *S*, and (**C**) *E*. The dotted lines between the x- and y-axis of the scatterplot indicate the reference Ct values of 20, 30, or 40. Each sample was tested using real-time PCR under the indicated conditions.

Among the 3990 participants, 26.0% (1039/3990) tested positive for the virus in the HCW-collection group, the self-collection group, or both groups. Self-collected samples had a higher detection rate than those in HCW-collected samples (HCW-collection (90.0%; 935/1039) versus self-collection (91.8%; 954/1039), paired *t*-test; *p* < 0.001) (Table 2). The Ct values for each gene (*N*, *RdRP* and *S*, and *E*) were compared in (81.8%; 850/1039) samples from the HCW-collection and self-collection groups that were positive for the virus (Figure 1). In the concordant positive group (samples that tested positive for the virus in both the HCW- and self-collection groups), more samples in the HCW-collection group had a low Ct value than those in the self-collection group (this was true for all the tested genes): *N* (82.0%, 697/850; *RdRP* and *S* (79.1%, 672/850); and *E* (79.5%, 676/850) (Table 2 and Figure 1). Overall, the results indicate that HCW-collected samples had a lower Ct value of SARS-CoV-2 than that of the self-collected samples.

Among the samples with discordant results (18.2%; 189/1039), 104 tested positive for the virus in the self-collection group but tested negative in the HCW-collection group. Eighty-five participants tested positive for SARS-CoV-2 in the HCW-collection group but tested negative in the self-collection group (Table 2). These findings imply that the self-collection method is reasonably sensitive, detecting > 90.9% of the SARS-CoV-2 infections diagnosed after sample collection by HCWs (Table 2). Our positive rates for the self-collected samples are comparable to those reported in other studies.

### 3.3. Clinical Performance of the HCW- and Self-Collection Methods

Cohen’s kappa analysis was performed to analyze the correlation between the two collection methods. For all three genes in the self-collection group, 21.3% (850/3990) tested positive for SARS-CoV-2. The sensitivity and specificity for the self-collection group were 90.9% (95% CI: 88.9–92.7) and 96.6% (95% CI: 95.9–97.2), respectively. In particular, the clinical performance of self-collected samples was in strong agreement and not statistically different from that of HCW-collected samples (κ = 0.87, McNemar’s test; *p* = 0.19) (Table 2).

### 3.4. Viral Load Detected Using the HCW- and Self-Collection Methods

**Figure 2:**
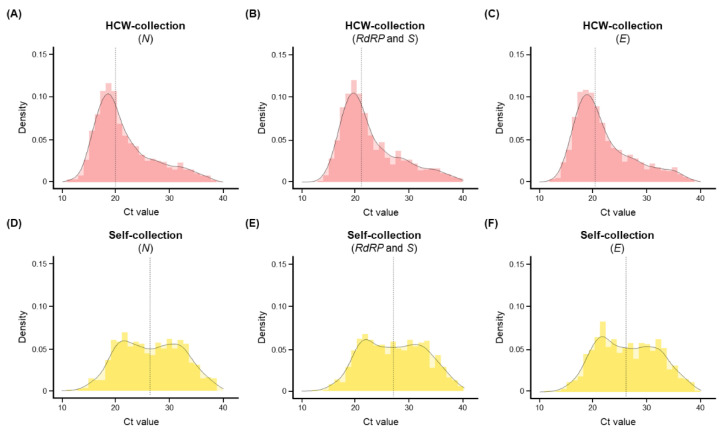
Histograms showing the distribution of Ct values for concordant positive specimens (*n* = 850). (**A**–**C**) Density of (**A**) *N*, (**B**) *RdRP* and *S*, and (**C**) *E* in the HCW-collection group. (**D**–**F**) Density of (**D**) *N*, (**E**) *RdRP* and *S*, and (**F**) *E* in the self-collection group. The y-axis of the histograms represents the estimation of the probability density estimation, whereas the vertical line presents the mean values.

As shown in Figure 2, the target viral genes in 850 individuals with concordant results were analyzed following immediate collection. Positive samples in the HCW-collection group had a mean Ct value of 21.6 (range: 17.6–24.4) for *N*, 22.7 (range: 18.8–25.6) for *RdRP* and *S*, and 21.9 (range: 18.1–25.0) for *E* (Figure 2A–C). In contrast, positive samples in the self-collection group had a mean Ct value of 26.6 (range: 21.8–31.3) for *N*, 27.1 (range: 22.3–31.6) for *RdRP* and *S*, and 26.3 (range: 21.6–30.7) for *E* (Figure 2D–F). The mean difference in Ct values ranged from 4.3 to 5.0, indicating that the viral load was 18.4–28.8 times lower in self-collected samples than in HCW-collected samples (Figure S1).

### 3.5. Comparison of Ct Values in the HCW-Collection and Self-Collection Groups

**Figure 3:**
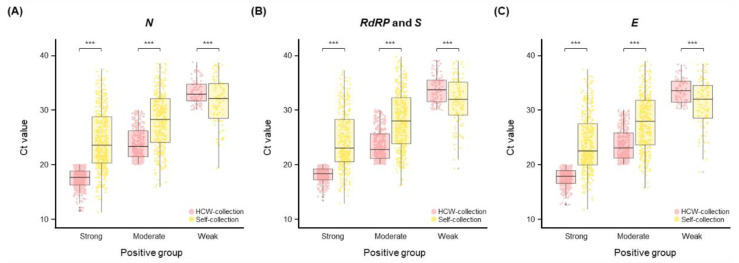
Distribution of the Ct values for SARS-CoV-2 in concordant positive specimens (*n* = 850). Boxplot of (**A**) *N*, (**B**) *RdRP* and *S*, and (**C**) *E*. The box plots present the median (interquartile range) values. *** *p* < 0.001.

We analyzed and compared the Ct values of self-collected and HCW-collected samples. The Ct values were as follows: strongly positive (median 17.7–18.3 for HCW-collection versus 22.5–23.6 for self-collection), moderately positive (median 22.7–23.3 for HCW-collection versus 27.9–28.2 for self-collection), and weakly positive (median 32.9–33.7 for HCW-collection versus 31.9–32.1 for self-collection) (Figure 3). Compared with self-collected samples, the HCW-collected samples had a lower Ct value for strongly and moderately positive samples. In contrast, the HCW-collected samples had a higher Ct value for weakly positive samples. All groups (strongly, moderately, and weakly positive) of Ct values differed significantly between HCW- and self-collected samples (paired *t*-test; *p* < 0.001).

## 4. Discussion

SARS-CoV-2 has frequently exhibited new mutations that have resulted in new variants, including Delta (B.1.617.2) and Omicron (B.1.1.529). Moreover, Omicron is evolving rapidly and its new subvariants are constantly emerging (e.g., BA.1, BA.2 and BA.5) [28]. The emergence of SARS-CoV-2 variants carrying mutations in the S gene raises concerns about the possibility of enhanced transmission during the ongoing COVID-19 pandemic [29]. As the pandemic spreads, with a rapidly increasing number of positive cases [30], the workload of HCWs becomes more physically exhausting, leading to pandemic fatigue [31]. NPS collection by HCWs is the conventional approach for SARS-CoV-2 testing [10]. However, the collection of NPS exposes frontline HCWs to the virus, whereas self-collection eliminates this risk and simultaneously processes the amount of sample collections needed, which is particularly advantageous given the current global health crisis [32].

It is crucial that samples are collected promptly so that communities can undergo testing for widespread infection without delay. Self-sampling is an excellent approach in these situations, as it allows for more samples to be collected and screened in less time. Self-collection of upper respiratory tract samples via the nasal cavity (such as NS) or oral cavity (such as saliva, OS, or mouthwash) is, therefore, being investigated as a minimally invasive technique, which poses less risk of infection to HCWs, and does not require the use of expensive personal protective equipment [10,11,33]. Thus, self-collection could be easily conducted at home or in the community in cases where close contact is required with patients having SARS-CoV-2 infection for rapid screening and detection of the virus during mass infection, thereby, eliminating close interaction of HCWs individuals with SARS-CoV-2 infection.

Some studies have reported low sensitivities of self-collected specimens (saliva or NS) from asymptomatic patients [11]. Saliva, in particular, has serious limitations in its usefulness due to its viscosity and the presence of various types of inhibitors [34]. In addition, different sampling sites can affect the early detection of SARS-CoV-2 [35,36,37]. Therefore, self-collected swabs were used in this study. This investigation was conducted according to the guidelines of the Korean Society for Laboratory Medicine guidelines and the Korea Centers for Disease Prevention and Control for the diagnosis of COVID-19 [25]. We enrolled 3990 eligible participants, who were provided with two swabbing methods (self-collection or HCW-collection). To avoid misdiagnosis, HCWs collected both NPS and OPS in the same viral transport medium. In addition, self-collected NS and OS samples were also combined in a transport medium.

The primary finding of this study was that there was a strong correlation between the performance of self- and HCW-collected swabs (κ = 0.87, McNemar’s test; *p* = 0.19). Moreover, the self-collection method demonstrated a sensitivity of 90.9 (88.9–92.7) and a specificity of 96.6 (95.9–97.2) (Table 2). These findings imply that self-collected swabs are as accurate as HCW-collected swabs for viral detection.

It has been suggested that SARS-CoV-2 can be detected in various specimens (such as NPS, OPS, saliva, urine, and stools) [9,10,38], and that the viral load in the upper respiratory tract is the highest during onset and in the last few days of symptoms [39]. However, it is not apparent whether samples from the upper respiratory tract have higher detection rates [40]. We found that SARS-CoV-2 detection rates were similar between self- and HCW-collected samples, although viral loads were lower in self-collected samples than in HCW-collected samples (Figure 3 and Supplementary Figure S1), as previously reported [10,41]. However, an analysis of weakly positive samples, namely, those with Ct > 30 (<10^5^ copies/mL), revealed a decreased viral load in the HCW-collection group (Figure 3). These results indicate that the HCW-collection method detects SARS-CoV-2 at a higher viral load than the self-collection method (Figure 2), despite the effect of several variables such as the sampling worker, individual proficiency, and time interval. Nevertheless, although the self-collection method shows a low viral load and varies with the self-sampling ability, it may be advantageous for early diagnosis of COVID-19. We also emphasize that the diagnostic capabilities between HCW- and self-collected samples are statistically similar, as a criterion for an accurate diagnosis of COVID-19.

This study has three limitations. First, the participants were selected based on their ability to collect samples suitable for analysis using an endogenous internal control. This is because it is difficult to assess if an exogenous internal control is appropriate for collection. Second, other respiratory specimens that can be obtained using the self-collection method, such as saliva, were not evaluated. Third, depending on the sampling site, viral load or clinical performance can affect the interpretation of the results. Therefore, additional research is required to investigate the effectiveness of different collection methods in participants with typical COVID-19 symptoms (such as cough and fever), compare detection rates in other specimens (such as saliva), and examine the effects of endogenous internal controls. Finally, because this was a retrospective study where we focused on the detection ability of the early-stage self-collection system, no separate verification procedure was undertaken because it was difficult to follow-up on the disease progression. However, we conducted a verification process for the inconsistent samples by repeating the test, from nucleic acid extraction to the mRT-qPCR assay. Therefore, future studies are needed to perform follow-up studies and test using other platforms, including rapid antigen tests or other assay kits.

## 5. Conclusions

**Figure 4:**
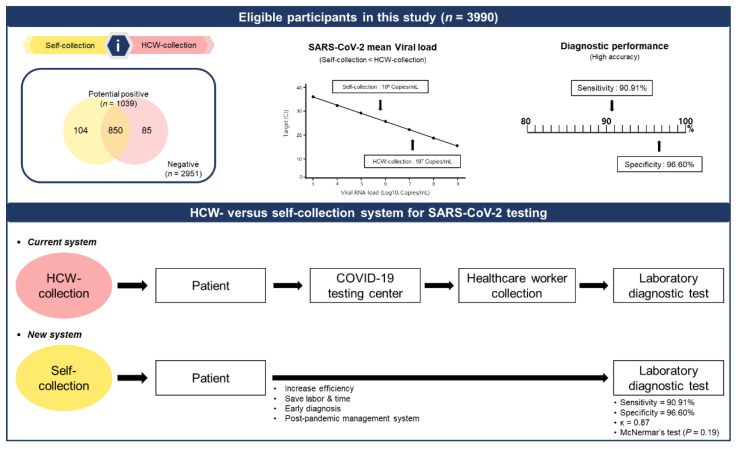
Summary of clinical performance and contribution of the self-collection system.

Owing to the global lifting of all COVID-19 restrictions and the emergence of highly infectious SARS-CoV-2 variants, such as Omicron, we believe that the sampling method needs to be highly reliable to manage community-based screening and necessary isolation. In our study, we validated two hypotheses: firstly, the SARS-CoV-2 viral load required for self-collection may be lower than that of HCW-collection; secondly, the sensitivities of self-collected NS and OS swabs for SARS-CoV-2 were comparable to those of HCW-collected NPS and OPS swabs, despite the viral load variation. The data presented support the notion that self-collection of swabs is an acceptable substitute for HCW-collection for mRT-qPCR analysis. In addition, self-collection reduces the exposure of HCWs to SARS-CoV-2 and can improve testing capacity during and after the COVID-19 pandemic (Figure 4).

## Figures and Tables

**Table 1:** Demography and characteristics of the study participants.

Demography/Characteristics	Self-Collection (*n* = 3990)		HCW-Collection (*n* = 3990)	
	Positive	Negative	Positive	Negative
Collected sample, n	954	3036	935	3055
Demography				
Age, years	22 (20–26)	24 (21–30)	22 (20–26)	24 (21–30)
Ct value				
*N*	27.6 (22.3–32.1)	N/A	20.3 (17.9–26.3)	N/A
*RdRP* and *S*	27.9 (22.6–32.5)	N/A	21.6 (19.1–27.7)	N/A
*E*	27.0 (22.0–31.9)	N/A	20.8 (18.2–26.6)	N/A

Data are presented as the median (interquartile range). Abbreviations: *N*, gene encoding the nucleocapsid protein; *RdRP*, gene encoding the RNA-dependent RNA polymerase; *S*, gene encoding the spike protein; *E*, gene encoding the envelope protein; N/A, not available.

**Table 2:** Comparison of clinical diagnosis performance between self-collection and HCW-collection.

	HCW-Collection		
	Positive	Negative	Total
**Self-collection**			
Positive, *n*	850	104	954
Negative, *n*	85	2951	3036
Total, *n*	935	3055	3990
Sensitivity, % (95% CI)	90.9 (88.9–92.7)		
Specificity, % (95% CI)	96.6 (95.9–97.2)		
Cohen’s kappa	0.87		
McNemar’s test *p*-value	0.19		

Abbreviations: CI, confidence interval; HCW, health care worker.

## Data Availability

All data are available within the article.

## Supplementary Materials

The following are available online at https://www.mdpi.com/article/10.3390/diagnostics12102279/s1, Supplementary Figure S1: Ten-fold dilutions ranging from 10^9^ to 10^3^ copies of SARS-CoV-2 RNA were tested in triplicates using real-time RT-PCR.
