# Supplementary material for: Performance Analysis of Self-Collected Nasal and Oral Swabs for Detection of SARS-CoV-2
Source: Diagnostics (Basel). 2022 Sep 21;12(10):2279. doi: 10.3390/diagnostics12102279 (PMC9600397; doi:10.3390/diagnostics12102279)
Supplement: Supplementary file 1 [file diagnostics-12-02279-s001.zip › diagnostics-1906431-supplementary.pdf]

Supplementary Results

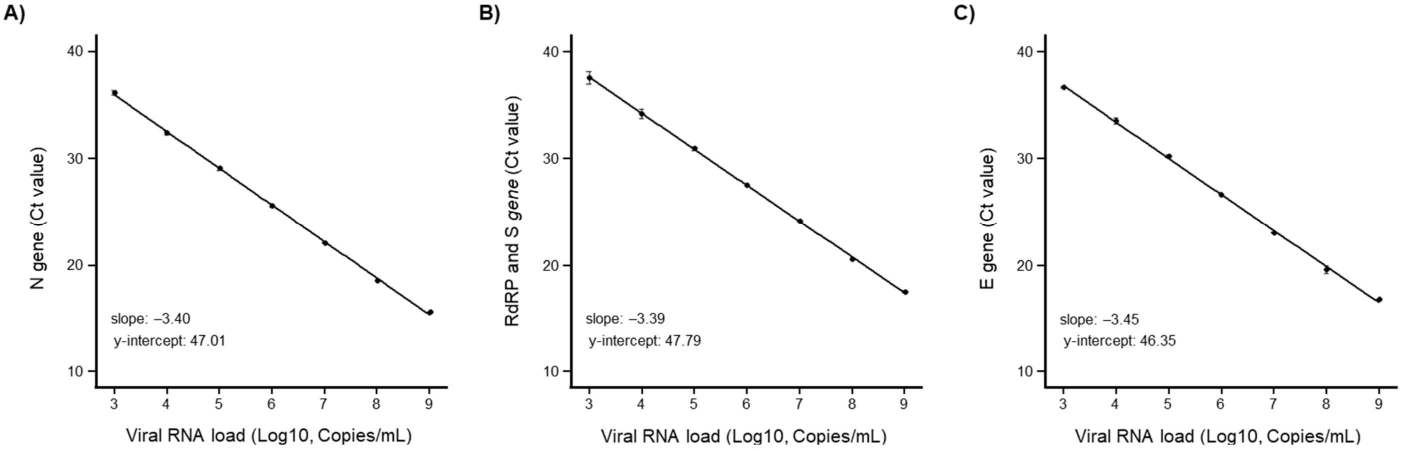

Supplementary Figure S1. Ten-fold dilutions ranging from 10^9 to 10^3 copies of SARS-CoV-2 RNA were tested in triplicates using real-time RT-PCR. A standard curve of the (A) *N*, (B) *RdRP* and *S*, and (C) *E* was generated by plotting the Ct values on the y-axis and the log10 of the input on the x-axis.
